# Quercetin improves diabetic kidney disease by inhibiting ferroptosis and regulating the Nrf2 in streptozotocin-induced diabetic rats

**DOI:** 10.1080/0886022X.2024.2327495

**Published:** 2024-03-11

**Authors:** Lei Zhang, Xingzhi Wang, Liang Chang, Yiqun Ren, Manshu Sui, Yuting Fu, Lei Zhang, Lirong Hao

**Affiliations:** a Department of Nephropathy and Hemodialysis, First Affiliated Hospital of Harbin Medical University, Harbin, Heilongjiang Province, China; b Department of Nephropathy, Southern University of Science and Technology Hospital, Shenzhen, China; c Department of Pathology, First Affiliated Hospital of Harbin Medical University, Harbin, Heilongjiang Province, China

**Keywords:** Diabetic kidney disease (DKD), quercetin, rat, ferroptosis, streptozotocin, Nrf2

## Abstract

Diabetic kidney disease (DKD) is a leading factor in end-stage renal disease. The complexity of its pathogenesis, combined with the limited treatment efficacy, necessitates deeper insights into potential causes. Studies suggest that ferroptosis-driven renal tubular damage contributes to DKD's progression, making its counteraction a potential therapeutic strategy. Quercetin, a flavonoid found in numerous fruits and vegetables, has demonstrated DKD mitigation in mouse models, though its protective mechanism remains ambiguous. In this study, we delved into quercetin’s potential anti-ferroptotic properties, employing a DKD rat model and high glucose (HG)-treated renal tubular epithelial cell models. Our findings revealed that HG prompted unusual ferroptosis activation in renal tubular epithelial cells. However, quercetin counteracted this by inhibiting ferroptosis and activating NFE2-related factor 2 (Nrf2) expression in both DKD rats and HG-treated HK-2 cells, indicating its renal protective role. Further experiments, both *in vivo* and *in vitro*, validated that quercetin stimulates Nrf2. Thus, our research underscores quercetin’s potential in DKD treatment by modulating the ferroptosis process *via* activating Nrf2 in a distinct DKD rat model, offering a fresh perspective on quercetin’s protective mechanisms.

## Introduction

Diabetes mellitus is a chronic metabolic disorder marked by high blood glucose levels, which over time can lead to various diabetic complications. Estimates suggest that the worldwide incidence of diabetes will increase from 10.5% (537 million people) in 2021 to 12.2% (783 million people) by 2045 [1]. The primary concern with diabetes mellitus is its tendency to lead to macrovascular and microvascular complications over time. Approximately one-third of people with diabetes are affected by diabetic kidney disease (DKD), which is a major cause of end-stage renal disease (ESRD) [2,3]. Diabetic kidney disease typically starts with microalbuminuria, progresses to macroalbuminuria, and then leads to a steady decline in kidney function. It is diagnosed by specific pathological changes, including an expanded mesangial matrix, nodular formations, and tubulointerstitial fibrosis [4]. The pathophysiology of DKD involves a complex interplay of factors such as metabolic and hemodynamic changes, oxidative stress, and activation of the renin-angiotensin system [5]. In addition to multidisciplinary approaches, sodium-glucose cotransporter 2 (SGLT2) inhibitors were introduced as a new preferred medication for DKD treatment in 2019 [6]. Despite treatment with renin-angiotensin system (RAS) and SGLT2 inhibitors, a proportion of patients still progress to end-stage kidney disease (ESKD), suggesting the involvement of alternative pathological processes in the development of DKD [7].

Ferroptosis is a recently identified form of non-apoptotic cell death characterized by iron-dependent lipid peroxidation [8]. Growing research indicates that ferroptosis plays a role in various human diseases, including those affecting the kidneys [9,10]. It is noteworthy that the kidney is particularly susceptible to iron-dependent ferroptosis. The deactivation of glutathione peroxidase 4 (GPX4), a key regulator of ferroptosis, leads to acute renal failure by initiating ferroptosis in the tubular cells of the kidneys in mice [11]. Recent research suggests that ferroptosis in renal tubular epithelial cells plays a role in the onset and advancement of diabetic kidney disease (DKD) [12]. Therefore, inhibiting ferroptosis has emerged as a potential therapeutic approach for managing DKD. Ferrostatin-1 (Fer-1), a small-molecule antioxidant that inhibits lipid peroxidation and ferroptosis, has been shown to reduce tubular damage in mice with DKD [13,14]. The limited *in vivo* stability of Ferrostatin-1 (Fer-1) hinders its practical application, highlighting the need to develop more stable and effective ferroptosis inhibitors [13].

Quercetin, a natural flavonoid found abundantly in vegetables and fruits, is noted for its stability and possesses anti-inflammatory, antiviral, and antioxidant properties [15]. Quercetin’s antioxidant action is primarily exhibited through multiple mechanisms, including boosting glutathione (GSH) levels, enhancing antioxidant signaling pathways, and counteracting oxidative damage from reactive oxygen species (ROS) [14,16]. Updated research suggests that quercetin can bind to iron, which may have a protective effect against ferroptosis in cases of acute kidney injury [17] and DKD as demonstrated in the db/db mouse model [14]. While the db/db mouse, with leptin deficiency, is a common model for studying DKD, it doesn’t fully mimic the disease’s later, more complex stages [18]. As an alternative, the Streptozotocin (STZ)-induced DKD rat model is less expensive and easier to induce [19]. Additionally, rats are more similar to humans in physiology, morphology, and genetics, which makes them particularly suitable for medical research and relatively easier to work with [20].

In this study, our goal was to assess the impact of quercetin on high glucose (HG)-induced damage in renal tubular epithelial cells and on DKD rat model, as well as to delve into the potential underlying molecular mechanisms. Our findings indicated that high glucose levels prompted the onset and progression of ferroptosis in the kidneys of rats with DKD. Quercetin helped to mitigate this diabetic kidney injury by obstructing ferroptosis, an effect achieved through the activation of Nrf2. Our research provides supporting evidence that quercetin has potential as a new candidate for the development of ferroptosis inhibitors in the treatment of diabetic kidney disease.

## Materials and methods

### Cell culture and treatment

Human kidney proximal tubular epithelial (HK-2) cells, sourced from Liaoning Changsheng Biotechnology Co., Ltd. in Benxi, were cultured in DMEM/F12 medium (Gibco, Thermo Fisher Scientific, Shanghai, China) supplemented with 5.5 mM glucose (normal control, CON), 20% fetal bovine serum (Gibco), and 1% antibiotics (penicillin/streptomycin, Procell, Wuhan, China). The cell incubation occurred at 37 °C in a 5% CO2 atmosphere. To establish an optimal high glucose (HG) environment for DKD *in vitro* model [21], HK-2 cells were incubated with varying glucose concentrations (5.5 mM, 17.5 mM, 30 mM, 45 mM, and 60 mM) for 48h, and at different incubation time points (0h, 12h, 24h, 48h, and 72h) under HG (30 mM) and CON (5.5 mM), with cell viability being assessed thereafter. Quercetin’s influence on HG-stressed HK-2 cells was examined by treating the cells with a range of quercetin concentrations (0 µM, 5 µM, 10 µM, 15 µM, 25 µM, and 50 µM). The chosen concentration of 25 µM quercetin was used for 48 h on HG-induced HK-2 cells (HG + QCT), with 24.5 mM mannitol as the osmotic control (HM), with Ferrostain-1 (10 μM, HG + Fer-1) serving as a positive control. HK-2 cells under normal glucose conditions (5.5 mM) acted as the baseline control (CON), and a subset of these cells were also treated with 25 µM QCT as an additional control (CON + QCT). To the investigate the Nrf2, the inhibitor ML-385 (5 µM) was administered to the QCT-treated, HG-stressed HK-2 cells. Subsequent to treatments, cells were collected for further experimental analyses.

### Cell viability assay

Cell viability was determined using a Cell Counting Kit-8 (CCK-8) detection Kit (CA1210, Solarbio, Beijing, China) according to the manufacturer’s protocol [22]. In brief, HK-2 cells were seeded into 96-well plates at a density of 3–4 × 10^3^ cells per well, and then incubated with 10 μL CCK-8 solution for 2 h at 37 °C, and then measured the absorbance at 450 nm. Viability was expressed as the absorbance ratio of treated cells to control cells.

### Animals and treatments

A total of 28 male Sprague-Dawley rats, aged 4–5 weeks and weighing approximately 160 ± 10 g, were acquired from Shanghai Slack Laboratory Animal Co., Ltd., China. These rats were housed in a specific pathogen-free (SPF) environment at the experimental animal center of the First Affiliated Hospital of Harbin Medical University. The facility was maintained at 22–25 °C with 50–60% humidity, featuring a 12-h light/dark cycle. After a week of acclimatization on a standard diet, 7 rats were selected randomly as the control group (CON), and the remaining 21 were subjected to a diabetic kidney disease (DKD) model induction using a revised method from prior studies [19,23].

To induce the DKD model, the 21 SD rats were administered intraperitoneal injections of a streptozotocin (STZ) solution (50 mg/kg in 0.05 M sodium citrate buffer) following a 12-h fasting period, for 5 consecutive days. The control group received equivalent volumes of sodium citrate buffer. Following this protocol, SD rats exhibiting STZ-induced diabetes and displayed hyperglycemia seven days post-STZ injections, with blood glucose levels exceeding 16.7 mmol/L (mM), were categorized into their respective groups [24,25]. One group was treated with saline as DKD group, another received 100 mg/kg of quercetin (QCT) orally gavage daily as DKD + QCT group, and the last group was administered 5 mg/kg of Ferrostatin-1 as a positive control, namely the DKD + Fer-1 group. The control group was given saline orally as an additional control (CON). Urine samples for urinary albumin and creatinine concentration assessment were collected over 24 h in metabolic cages. During this period, the rats had unrestricted access to food and water, and care was taken to minimize any stressors that might affect the outcome. Post 12 weeks of treatment, blood samples were collected, and all rats were euthanized for kidney tissue preservation and analysis. This animal study was approved by the Harbin Medical University’s animal use committee (SYSK 2020-116), ensuring all methods were performed in line with the relevant guidelines and regulations.

### Histopathological study

Kidney tissue samples of rats were first fixed using 4% formaldehyde solution for an overnight period. Following fixation, the tissues were embedded in paraffin blocks. Sections measuring 3 μm in thickness were then sliced from these paraffin-embedded blocks. The sections were deparaffinized using xylene and went through a rehydration process. For histopathological examination, the rehydrated tissue sections were stained using Hematoxylin and Eosin (H&E) for general tissue structure visualization, Periodic Acid-Schiff (PAS) to highlight polysaccharides such as glycogen and structural carbohydrates. After staining, the specimens were examined and images were captured using a light microscope for detailed histological analysis.

### Immunohistochemical staining

Prior to staining, kidney sections from rats underwent deparaffinization in xylene, which clears the paraffin from the sections, followed by a graded rehydration process. This typically involves a series of alcohol baths of decreasing concentration to gently reintroduce water into the tissues. Then the rehydrated sections were then incubated with the following primary antibodies for targeted detection of proteins: GPX-4 (1:100, rabbit monoclonal, ab125066, Abcam), xCT (1:200, rabbit monoclonal, ab175186, Abcam), ACSL4 (1:200, rabbit monoclonal, MA5-42523, Invitrogen), and Nrf-2 (1:100, rabbit polyclonal, PA5-88084, Invitrogen). The primary antibody incubation was performed in a controlled environment at 4 °C overnight to allow for maximum binding specificity and efficiency. Following primary antibody incubation, the sections were washed to remove unbound antibodies, and incubated with horseradish peroxidase (HRP)-conjugated secondary antibody. Finally, the HRP-DAB system (Proteintech, Wuhan, China) was applied to visualize the sections, followed by staining with hematoxylin to detect HRP activity. All sections were imaged under an Olympus BX53F microscope.

### Measurement of lipid ROS by flow cytometry

The lipid ROS levels in HK-2 cells were determined by utilizing the FITC-BODIPY (Alexa Fluor 488), based on a previously described method [14,26]. This technique relies on the alteration of fluorescence characteristics of the dye when oxidized by lipid ROS. In summary, HK-2 cell groups under investigation were prepared as single cell suspensions, rinsed with PBS, and then incubated with 0.5 µM of FITC-BODIPY in 1 mL of PBS for 15 min at 37 °C. The fluorescence intensity was recorded on a Fortessa LSRII, using the B530 channel. To assess cell mortality, DRAQ7 (0.5 µM) (Biostatus; # DR71000) was introduced before collecting the data. FlowJo 10.7 software was utilized for the data analysis. The internal buildup of ROS was evaluated employing 20,70-dichlorodihydrofluorescein diacetate (DCFH-DA) (CA140, Solarbio, Beijing, China) as per the provided guidelines. ImageJ 8.0 software (NIH, USA) was used to measure the relative fluorescence intensity.

### Determination of iron content, glutathione (GSH) and lipid peroxides

The concentrations of iron, GSH, and lipid peroxides in the harvested HK-2 cells and kidney samples were analyzed independently utilizing specific assay kits: an Iron Assay Kit (MAK025, Sigma, USA) for iron, a Micro Reduced GSH Assay Kit (BC1175, Solarbio, Beijing, China) for GSH, and a Micro malondialdehyde (MDA) Assay Kit (BC0020, Solarbio, Beijing, China) for lipid peroxides. Each analysis was conducted strictly following the protocols provided by the manufacturers.

### Transmission electron microscopy (TEM)

The HK-2 cells that were collected underwent fixation with a solution containing 0.25% trypsin-EDTA and 1% osmic acid in 0.1 M phosphate-buffered saline (PBS) with a pH of 7.4. Following fixation, the samples were stained using lead citrate and a 2% solution of uranium acetate in water for contrast enhancement. The samples were then rinsed with PBS and dehydrated using acetone before being embedded in an epoxy resin matrix for stabilization. The final step involved preparing transmission electron micrographs with a transmission electron microscope (JEOL USA, Inc., Peabody, MA, USA) at an acceleration voltage of 80 kV to examine the fine structural details of the cells.

### Quantitative real-time polymerase chain reaction (qRT-PCR)

Total RNA was extracted from the gathered kidney tissues and cells using the RNeasy Mini Kit (Qiagen, Hilden, Germany), and then converted into cDNA with the help of the RevertAid First Strand cDNA Synthesis Kit (Thermo Fisher Scientific, Shanghai, China). The reverse transcription process involved the following thermal cycling conditions: an initial denaturation at 95 °C for 3 min, followed by 30 cycles that included denaturation at 95 °C for 30 s, annealing at 58 °C for 30 s, extension at 72 °C for 30 s, and a final extension at 4 °C for 30 min. β-actin served as the internal reference for normalization, and all primers used in this research (referenced as Table 1) were crafted and provided by Sangon Biotech (Shanghai, China). Quantitative real-time PCR (qRT-PCR) was employed to determine the relative expression levels of the target genes, utilizing Fast Start Essential DNA Green Master Mix (Roche, China), with calculations based on the 2^(-ΔΔCq) method.

**Table 1.** Primer sequences for qRT-PCR.

Gene (mouse)	Forward Primer (5’-3’)	Reverse Primer (5’-3’)
β-actin	TGGAATCCTGTGGCATCCAT	GCTAGGAGCCAGAGCAGTAA
GPX-4	GAGGCAAGACCGAAGTAAACTAC	CCGAACTGGTTACACGGGAA
ACSL4	CATCCCTGGAGCAGATACTCT	TCACTTAGGATTTCCCTGGTCC
xCT	TCTCCAAAGGAGGTTACCTGC	AGACTCCCCTCAGTAAAGTGAC
Nrf2	TCAGCGACGGAAAGAGTATGA	CCACTGGTTTCTGACTGGA

### Western blot

Proteins were isolated from the collected cells and kidney samples using a radio-immunoprecipitation assay (RIPA) lysis buffer (R0010, Solarbio, Beijing, China). Following extraction, the protein levels were determined using a Bicinchoninic Acid (BCA) protein assay kit (PC0020, Solarbio, Beijing, China). The western blot analysis was conducted according to established methods. Primary antibodies utilized included GPX-4 (1:1000 dilution, ab125066, rabbit monoclonal, Abcam), xCT (1:1000 dilution, ab175186, rabbit monoclonal, Abcam), ACSL4 (1:1000 dilution, MA5-42523, rabbit monoclonal, Invitrogen), and Nrf-2 (1:1000 dilution, PA5-88084, rabbit polyclonal, Invitrogen). Secondary antibodies, used at a 1:5000 dilution, were acquired from Proteintech (Wuhan, China).

### Statistical analysis

All results are presented as the average value plus or minus the standard deviation (SD). To determine statistical significance between two distinct groups, the Student’s t-test was utilized. For comparisons involving more than two groups, one-way Analysis of Variance (ANOVA) followed by Tukey’s *post hoc* test was employed. A *P*-value of less than 0.05 is considered to indicate a statistically significant difference.

## Results

### Quercetin improved renal function and alleviated renal tissue injury in DKD rats

**Figure 1.**
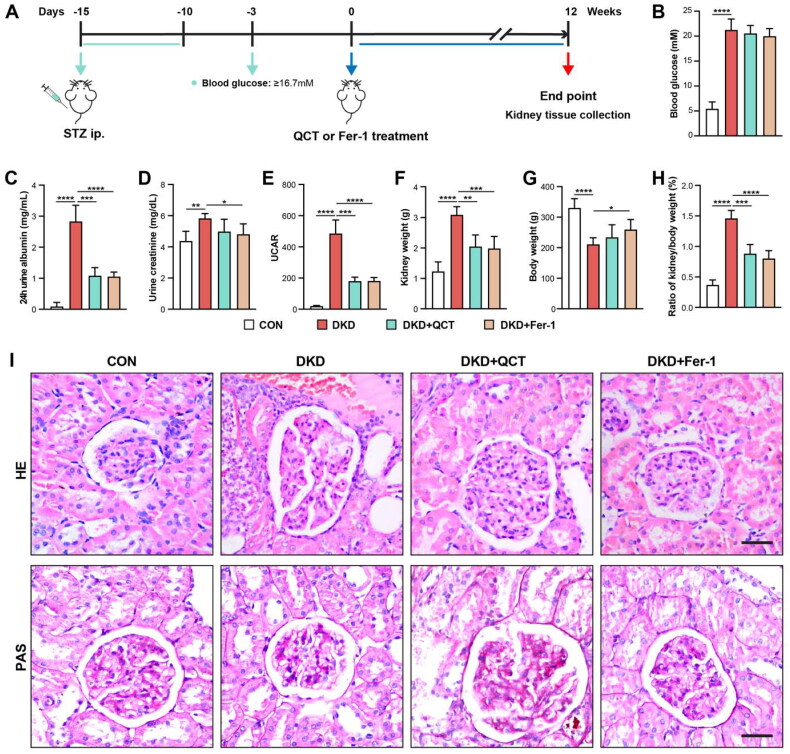
Quercetin’s effects on DKD rats’ renal function and tissue injury. (A) Diabetic kidney disease (DKD) rat model establishment with STZ and treatment regimen. (B) Post-12-week blood glucose level following quercetin or Fer-1 administration. Measurements at 12 weeks included urine albumin (C), creatinine (D), and UCAR (E). Kidney weight (F), body weight (G), and kidney/body weight ratio (H) were also assessed. (I) Kidney sections stained with HE (upper), and PAS (bottom) across groups (scale bar = 50 μm). Groups: CON (control), DKD (model), DKD + QCT (quercetin-treated), DKD + Fer-1 (Ferrostatin-1-treated). Mean data ± SD are shown. Significance levels: ****p < 0.0001; ***p < 0.001; **p < 0.01; *p < 0.05 *via* one-way ANOVA.

We established a DKD model in rats using streptozotocin (STZ). Male SD rats received STZ intraperitoneally at 50 mg/kg for five days. On the 7th day after STZ treatment, we measured blood glucose (Figure 1(A)), defining rats with levels above 16.7 mM as DKD models. These DKD rats were then split into three groups for a 12-week treatment: saline (DKD), quercetin (DKD + QCT), and Ferrostatin-1 (DKD + Fer-1), with healthy (non-DKD) rats as controls (CON) (Figure 1(A)). After 12 weeks, DKD rats exhibited higher blood glucose levels compared to the controls (Figure 1(B)). Quercetin and Fer-1 treatments resulted in minor, non-significant reductions in glucose levels in DKD rats (Figure 1(B)). The DKD rats also developed higher levels of albumin, creatinine and UACR, indicating successful model establishment (Figure 1(C–E)). Quercetin and Fer-1 significantly decreased these markers, suggesting a nephroprotective effect. The kidney weight, body weight and kidney/body weight ratio of rats further confirmed model efficacy and treatments benefits (Figure 1(F–H)). Kidney biopsies, stained with HE, and PAS (Figure 1(I)), showed less mesangial matrix and renal tubular injury in DKD rats post-treatment, highlighting the therapeutic effects of quercetin and Fer-1 on kidney health in DKD rats.

### Quercetin alleviated kidney injury in DKD rats by suppressing ferroptosis

**Figure 2.**
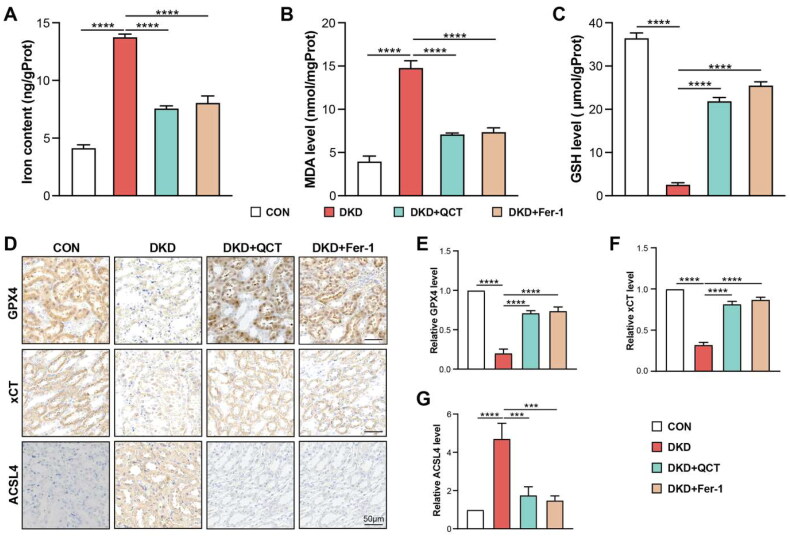
Quercetin mitigates ferroptosis-induced kidney damage in DKD rats. (A) Iron, MDA (B), and GSH (C) levels in rat kidney tissues were determined using biochemical assays. (D) Immunohistochemistry for GPX4, xCT, and ACSL4 in rat kidneys (scale bar = 50 μm). Quantitative analysis of GPX4 (E), xCT (F), and ACSL4 (G) expression from Figure 2(D). Groups: CON (control), DKD (DKD model), DKD + QCT (DKD rats treated with Quercetin), DKD + Fer-1 (DKD rats treated with Ferrostatin-1). Data represent means ± SD. Significance: ****p < 0.0001; ***p < 0.001, assessed by one-way ANOVA.

As ferroptosis is dependent upon intracellular iron and occurs due to lipid peroxide accumulation [27], we assessed the intracellular iron, GSH and MDA levels in rat kidney tissues. DKD rats had higher iron and MDA levels, with reduced GSH compared to controls (CON), indicating the oxidative stress disturbance in DKD rat model (Figure 2(A–C)). Quercetin and Fer-1 treatments decreased iron, increased GSH, and lowered MDA levels, suggesting their nephroprotective effects (Figure 2(A–C)). The ferroptosis regulator, regulator glutathione peroxidase 4 (GPX4), crucial for inhibiting lipid peroxidation [28], was analyzed *via* immunohistochemistry (IHC) in kidney slices. GPX4 was predominantly found in the kidney tubulointerstitium of healthy control rats, particularly in tubular epithelial cells (Figure 2(D)), but was significantly reduced in DKD rats. This reduction was mitigated by treatments with QCT and Fer-1 (Figure 2(E)). Previous studies indicated that xCT and acyl-CoA synthetase long-chain family member 4 (ACSL4) are the key mediators of ferroptosis during diabetic kidney injury [12,29]. IHC staining revealed decreased xCT and increased ACSL4 expression in DKD rats relative to controls, changes that were significantly reversed by quercetin and Fer-1 treatments (Figure 2(D, F, G)), highlighting their role in managing ferroptosis and diabetic kidney injury.

### Quercetin suppressed ferroptosis by activating the Nrf2

**Figure 3.**
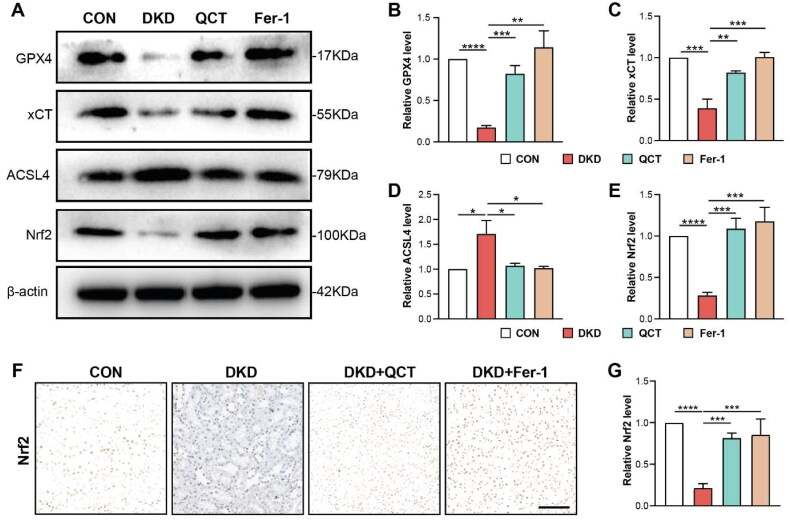
Quercetin activates Nrf2 to inhibit ferroptosis in DKD rats. (A) Western blot analyses of GPX4, xCT, ACSL4, and Nrf2 protein levels in kidney tissues. (B,D) Semi-quantitative assessments of GPX4, xCT, and ACSL4 from immunoblots. (E) Semi-quantitative analysis of Nrf2 from immunoblots. (F) Immunohistochemical staining for Nrf2 in kidney tissues (scale bar = 50 μm). (G) Quantification of Nrf2 expression from Figure 3(F). Groups: CON (control), DKD (DKD model), DKD + QCT (DKD rats treated with Quercetin), DKD + Fer-1 (DKD rats treated with Ferrostatin-1). Data are mean ± SD. Significance levels: ****p < 0.0001; ***p < 0.001; **p < 0.01; *p < 0.05, determined by one-way ANOVA.

Western blot analysis was conducted to investigate the *in vivo* effect of quercetin on ferroptosis by examining the expression of GPX4, xCT, and ACSL4, crucial ferroptosis regulators. In DKD rats, the GPX4 and xCT protein levels were significantly reduced, while ACSL4 levels increased, confirming our IHC results (Figure 3(A–D)). Quercetin and Fer-1 treatments notably upregulated GPX4 and xCT, and downregulated ACSL4, aligning these levels more closely with those of control rats, indicating a ferroptosis-inhibiting effect by quercetin in renal tubular epithelial cells (Figure 3(A–D)). The transcription factor, erythroid 2-related factor 2 (Nrf2), a key regulator of the cellular antioxidant response, pivotal in regulating ROS and preventing cell ferroptosis, was assessed [30]. DKD rats showed decreased Nrf2 expression in kidney tissues compared to controls, as revealed by Western blot and immunohistochemistry (Figure 3(A, E, F, G)). Treatments with quercetin or Fer-1 successfully restored Nrf2 expression in DKD rats (Figure 3(E, F, G)). These findings suggest that quercetin mitigates ferroptosis-induced kidney injury in DKD rats by activating the Nrf2.

### Quercetin alleviated HG-induced damage in renal tubular epithelial cells

**Figure 4.**
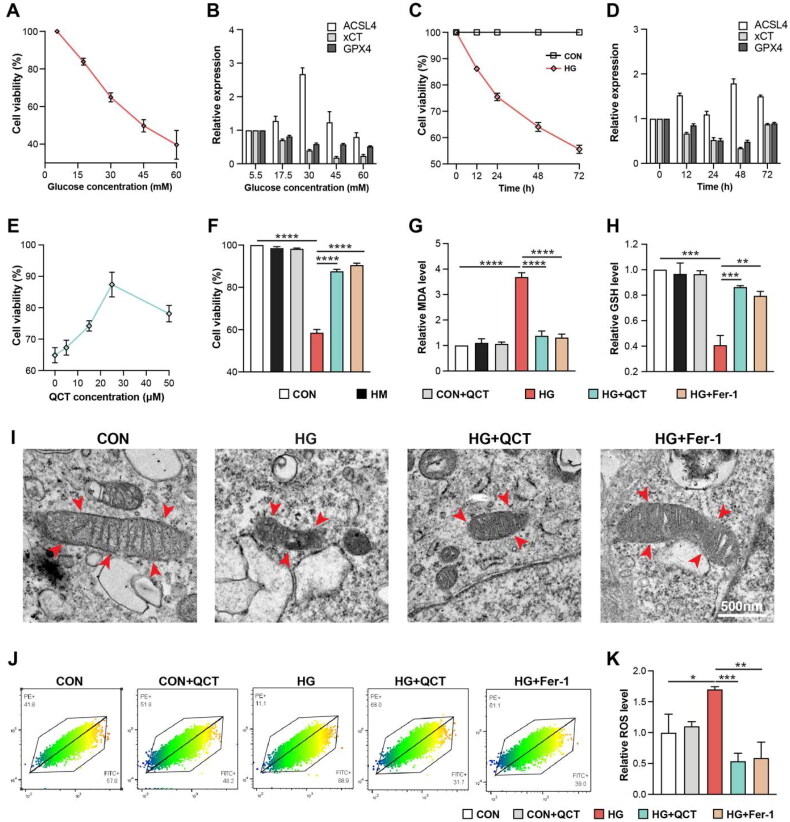
Quercetin mitigates high glucose-induced damage in renal tubular epithelial cells. (**A**) HK-2 cell viability across various glucose concentrations (5.5 mM, 17.5 mM, 30 mM, 45 mM and 60 mM) assessed *via* CCK-8 assay. (**B**) The mRNA expression levels of ACSL4, GPX4, and xCT in HK-2 cells after treatment with varying glucose concentrations in Figure 4A. (**C**) Viability of HK-2 cells in high-glucose (HG, 30 mM) and control (CON, 5.5 mM) conditions over different incubation time points (0, 12h, 24h, 48h, 72h) measured by CCK-8 assay. (**D**) The mRNA expression levels of ACSL4, GPX4, and xCT in HK-2 cells exposed to high-glucose (30 mM) and control (CON, 5.5 mM) conditions over different incubation time in Figure 4C. (**E**) CCK-8 assay determining HK-2 cell viability post-quercetin treatment at different doses (0, 5 μM, 15 μM, 25 μM, 50 μM). (**F**) Cell viability comparison among HK-2 cells groups *via* CCK-8 assay. The MDA (**G**) and GSH (**H**) levels in HK-2 cells groups quantified using biochemical assays. (**I**) Transmission electron microscopy (TEM) used to observe mitochondrial ultrastructural changes in HK-2 cells (scale bar = 500 nm), with mitochondria indicated by red arrows. (**J**) Flow cytometry (FACS) quantification of reactive oxygen species (ROS) levels in HK-2 cell groups. (**K**) The ROS expression level in Figure 4(J). CON: control HK-2 cells; HM: control HK-2 cells receiving mannitol treatment; CON + QCT: control HK-2 cells receiving quercetin treatment; HM: HG: HK-2 cells under high-glucose (HG); HG + QCT: HK-2 cells under HG incubation receiving quercetin treatment; HG + Fer-1: HK-2 cells under HG incubation receiving Fer-1 treatment. Data are mean ± SD. Significance levels are indicated as follows: ****p < 0.0001; ***p < 0.001; **p < 0.01; *p < 0.05, determined by one-way ANOVA.

To investigate the *in vitro* effects of quercetin on diabetic kidney disease (DKD), HK-2 cells were exposed to varying glucose concentrations (5.5 to 60 mM), simulating the microenvironment of DKD. Higher glucose levels linearly decreased cell viability (Figure 4(A)). At 30 mM glucose, a marked reduction in HK-2 cell viability was observed over time, in contrast to stable viability in untreated cells (Figure 4(C)). Subsequent assessment of high glucose’s (HG) effect on ferroptosis involved evaluating the expression levels of GPX4, xCT, and ACSL4. Notably, HG treatment led to an increase in ACSL4 expression and a decrease in GPX4 and xCT levels, with the most significant changes occurring at 30 mM glucose (Figure 4(B)) and after 48 h of incubation (Figure 4(D)). In summary, to emulate the DKD condition *in vitro*, HK-2 cells were exposed to 30 mM glucose for 48 h, a setting that effectively induced ferroptosis. The efficacy of quercetin on HK-2 cells under high-glucose (HG) conditions was assessed by administering varying quercetin concentrations (0 to 50 μM). Optimal cell viability was observed at a quercetin concentration of 25 μM (Figure 4(E)). Post 48-h HG incubation at 30 mM, there was a notable decline in cell viability within the HG group when compared to control groups (Figure 4(F)). This reduction in viability was significantly ameliorated in the quercetin-treated group (Figure 4(F)). To confirm if ferroptosis was responsible for the viability decrease under HG, cells were treated with Fer-1, which demonstrated comparable protective outcomes to the quercetin treatment. HG conditions induced elevated malondialdehyde (MDA) levels and decreased glutathione (GSH) in HK-2 cells, deviations which were normalized upon treatment with quercetin or Fer-1 (Figure 4(G, H)). Ultrastructural analysis *via* transmission electron microscopy (TEM) revealed that treating HK-2 cells with 30 mM high glucose for 48 h significantly led to mitochondrial crista reduction, disappearance, and rupture of the outer membrane – key morphological changes indicative of ferroptosis (Figure 4(I)). However, these mitochondrial injuries were clearly restored after QCT and Fer-1 treatment (Figure 4(I)). Reactive oxygen species (ROS) were monitored *via* BODIPY intensity using flow cytometry (FACS), revealing significant increases post-HG incubation, which QCT and Fer-1 treatments successfully mitigated (Figure 4(J, K)). These findings suggest that QCT can mitigate HG-induced injury in renal tubular epithelial cells.

### Quercetin alleviated aberrant ferroptosis activation in HK-2 cells

**Figure 5.**
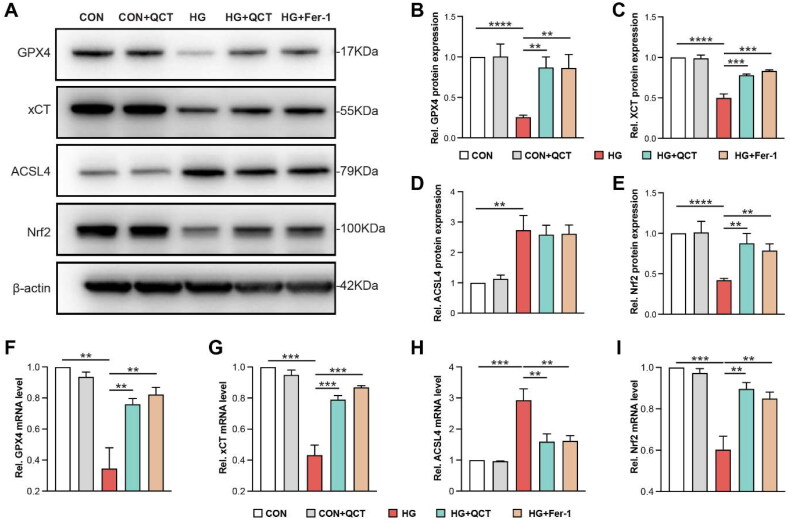
Quercetin alleviated aberrant ferroptosis activation in HK-2 cells. (**A**) Western blot analyses of GPX4, xCT, ACSL4, and Nrf2 protein levels in each group of HK-2 cells. (**B–E**) Semi-quantitative assessments of GPX4, xCT, ACSL4, and Nrf2 from immunoblots. (**F–I**) Quantitative PCR results showing relative mRNA expressions of GPX4, xCT, ACSL4, and Nrf2 in the cells from each group. CON: control HK-2 cells; CON + QCT: control HK-2 cells receiving quercetin treatment; HG: HK-2 cells under HG incubation; HG + QCT: HK-2 cells under HG incubation receiving quercetin treatment; HG + Fer-1: HK-2 cell under HG incubation receiving Fer-1 treatment. Data are mean ± SD. Significance levels are indicated as follows: ****p < 0.0001; ***p < 0.001; **p < 0.01; *p < 0.05, determined by one-way ANOVA.

To investigate the influence of quercetin on ferroptosis *in vitro*, we assessed the expression levels of GPX4, xCT, and ACSL4, key regulators of ferroptosis, by using Western blot and qPCR. In high glucose (HG)-stimulated HK-2 cells, GPX4 and xCT proteins and mRNA levels were notably reduced, whereas ACSL4 expression was increased (Figure 5(A–D, F–H)). These findings align with *in vivo* observations. Treatment with ferrostatin-1 (Fer-1) or QCT reversed these effects, elevating GPX4 and xCT levels toward those in the control (CON) group and diminishing ACSL4 expression (Figure 5(A–D, F–H)), indicating quercetin’s potential in mitigating ferroptosis in renal tubular epithelial cells. Moreover, the Nrf2, crucial for ROS regulation and cellular redox balance, showed diminished protein and mRNA levels in HG-challenged HK-2 cells compared to the CON group (Figure 5(A, E, I)). Both Fer-1 and QCT treatments were effective in restoring Nrf2 expression (Figure 5(A, E, I)). These results strongly suggest that QCT can ameliorate ferroptosis-driven renal injury in HK-2 cells by activating the Nrf2.

### *QCT protected HK-2 cells against ferroptosis* via *Nrf2*

**Figure 6.**
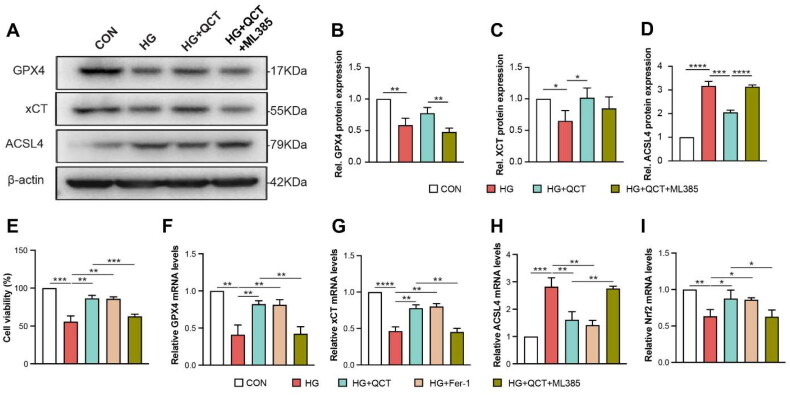
QCT protected HK-2 cells against ferroptosis *via* Nrf2. (**A**) Western blot analyses of GPX4, xCT and ACSL4 in each group of HK-2 cells. (**B–D**) Semi-quantitative assessments of GPX4, xCT, and ACSL4 from immunoblots. (**E**) HK-2 cell viability across various treatment groups (CON, HG, HG + QCT, HG + Fer-1 and HG + QCT + ML385) assessed *via* CCK-8 assay. (**F–I**) Quantitative PCR results showing relative mRNA expressions of GPX4, xCT, ACSL4, and Nrf2 in the cells from each group. CON: control HK-2 cells; HG: HK-2 cells under HG incubation; HG + QCT: HK-2 cells under HG incubation receiving quercetin treatment; HG + QCT + ML385: HK-2 cell under HG incubation receiving quercetin and ML385 treatment. Data are mean ± SD. Significance levels are indicated as follows: ****p < 0.0001; ***p < 0.001; **p < 0.01; *p < 0.05, determined by one-way ANOVA.

To elucidate the role of Nrf2 in quercetin’s protective effects against high glucose (HG)-induced injury in HK-2 cells, we employed the Nrf2 inhibitor, ML385. Our findings indicate that the protein and mRNA levels of GPX4 and xCT were significantly diminished, while the ACSL4 expression was notably increased in HG-induced HK-2 cells (Figure 6(A–D, F–H)), corroborating *in vivo* data. Treatment with QCT markedly enhanced the expression of GPX4 and xCT, aligning them with levels observed in the control (CON) group, and significantly reduced the expression of ACSL4 (Figure 6(A–D, F–H)). In addition, the reduction of cells viability in HG group was significantly ameliorated after the QCT treatment (Figure 6(E)). These results suggest that quercetin can inhibit ferroptosis in renal tubular epithelial cells. However, the addition of ML385 significantly negated quercetin’s protective effects in HG-induced HK-2 cells, evidenced by the decrease in GPX4 and xCT levels, an increase in ACSL4 expression, and reduction of cell viability (Figure 6(A–D, E–H)). Moreover, ML385 treatment substantially reduced Nrf2 mRNA levels (Figure 6(I)). Collectively, these outcomes indicate quercetin’s protective mechanism against HG-induced injury in HK-2 cells is likely through the inhibition of ferroptosis mediated by the Nrf2.

## Discussion

Diabetic kidney disease (DKD) represents a severe and common complication associated with diabetes, marked by increased levels of albumin in the urine and a gradual decline in kidney function due to the death of tubular cells [31]. The underlying causes of DKD are not yet fully understood, highlighting the critical need for in-depth research into its pathogenesis and the creation of effective therapeutic strategies. Quercetin is known for its strong antioxidant capabilities, which encompass neutralizing free radicals, chelating metal ions, inhibiting lipid peroxidation, and enhancing the activity of antioxidant enzymes [16]. Quercetin has been effective in guarding against oxidative stress, supporting the regeneration of pancreatic β-cells, improving insulin secretion, and reducing insulin resistance [32,33], and restored normal endothelial function in diabetic rats when used in conjunction with metformin [34]. Furthermore, quercetin may play a role in preventing and alleviating complications related to diabetes, such as diabetic kidney disease (DKD), though its exact mechanisms of action are still to be fully understood unclear [35]. Recent research has highlighted the involvement of ferroptosis, a form of regulated cell death, in the damage to renal tubular cells observed in DKD. Inhibiting ferroptosis has emerged as a novel approach for the treatment of DKD, suggesting a potential pathway through which quercetin could exert its protective effects against this condition [12,14,24,36–39]. Quercetin (QCT), a significant antioxidant, has been shown to offer protection against renal tubular epithelial cell death induced by ferroptosis. This suggests that quercetin possesses potential as a ferroptosis inhibitor, contributing to the repair of kidney damage [17]. In this study, we aimed to assess the effectiveness of quercetin as a ferroptosis inhibitor in the management of diabetic kidney disease (DKD) and to delve into the underlying molecular mechanisms involved.

To investigate the therapeutic potential of quercetin on the progression of diabetic kidney disease (DKD), we established a DKD model in rats through the administration of streptozotocin, inducing diabetes. Notably, quercetin administration led to reductions in blood glucose levels, the urine albumin-to-creatinine ratio (UACR), and weight loss, alongside enhancements in renal function in the DKD rats. Pathological examinations and immunohistochemical analyses revealed that quercetin markedly ameliorated kidney functionality by curtailing extracellular matrix (ECM) expansion and tubular damage. Furthermore, our findings demonstrated that quercetin significantly curtailed ferroptosis, as evidenced by decreased iron and malondialdehyde (MDA) levels, and elevated glutathione (GSH) levels. It was also observed that quercetin treatment significantly upregulated the expression of ferroptosis inhibitory proteins, such as GPX4 and xCT, while downregulating the pro-ferroptosis regulator ACSL4. *In vitro* assays involving high glucose (HG)-induced HK-2 cell injury models further supported these findings, with quercetin treatment improving cell viability and modulating the expression of key ferroptosis markers, including GPX4, xCT, and ACSL4. HK-2 cells under HG stress showed ferroptosis-like alterations, such as mitochondrial condensation and loss of mitochondrial cristae, alongside increased iron, and MDA levels, reduced GSH, and dysregulated expression of ferroptosis regulators. However, quercetin was effective in mitigating HG-induced HK-2 cell damage and reducing reactive oxygen species (ROS) production. Additionally, treatment of HK-2 cells under HG stress with ferrostatin-1 (Fer-1) or quercetin not only reversed iron overload and lipid peroxidation but also reduced cell death. Quercetin exhibited superior efficacy in inhibiting ferroptosis compared to Fer-1, aligning with recent findings on quercetin’s role in attenuating acute kidney injury (AKI) through ferroptosis inhibition [17].

Nrf2, a critical transcription factor, has been widely recognized not only as a pivotal gene in antioxidant defense mechanisms but also as an essential mediator in the process of ferroptosis [30]. The upregulation of Nrf2 can provide protection against cell damage induced by ferroptosis [40]. Recent studies have shown that the activation of Nrf2 plays a significant role in influencing the progression of endothelial injury and acute kidney injury by regulating oxidative stress and lipid peroxidation [41,42]. Activating the Nrf2 significantly enhances the expression of anti-oxidant enzymes, effectively reducing the production of intracellular reactive oxygen species (ROS) and decreasing iron accumulation within cells [43]. It has been previously reported that reduced expression of Nrf2 is associated with the development of diabetic kidney disease (DKD), suggesting a protective role of Nrf2 in kidney health in the context of diabetes [44]. Activating the Nrf2 signaling pathway has been shown to significantly mitigate diabetic kidney injury by inhibiting ferroptosis in renal tubular epithelial cells, highlighting its therapeutic potential in protecting renal function in the context of diabetes [14,24,39]. Current research suggests that high glucose (HG) conditions facilitate ferroptosis in HK-2 cells by inhibiting the Nrf2 signaling pathway, leading to an increase in intracellular iron and accumulation of lipid peroxides in rats with diabetic kidney disease (DKD). Furthermore, numerous studies have documented that quercetin (QCT) can safeguard against cell damage by mitigating oxidative stress through the modulation of the Nrf2 signaling pathway [36]. In this research, findings demonstrated that quercetin (QCT) notably decreased the concentration of intracellular iron and lipid peroxidation through the activating Nrf2. Moreover, experiments involving Nrf2 gene complementation revealed that the Nrf2 inhibitor, ML385, effectively triggered cell ferroptosis and diminished the nephroprotective effects of quercetin in rats with diabetic kidney disease (DKD), underscoring the pivotal role of the Nrf2 signaling pathway in quercetin’s mechanism of action against DKD progression. Thus, both *in vitro* and *in vivo* studies suggest that quercetin presents a viable therapeutic approach for DKD, especially by targeting the Nrf2 to robustly inhibit ferroptosis in renal tubular epithelial cells in a dose-dependent manner, showcasing its potential as an effective treatment strategy for DKD.

## Conclusions

This study conclusively underscores the nephroprotective capability of quercetin in treating diabetic kidney disease (DKD) by inhibiting ferroptosis in a rat model. The findings highlight the role of high glucose (HG) conditions in triggering abnormal activation of ferroptosis and establish quercetin as an effective ferroptosis inhibitor capable of mitigating kidney damage in DKD. This is achieved by reducing reactive oxygen species (ROS) levels and activating Nrf2, which plays a crucial role in mediating cellular ferroptosis. Our research offers compelling evidence that quercetin could act as a ferroptosis inhibitor, providing a novel approach to slow down the progression of DKD using rat model. The data presented herein mark an initial step toward developing a ferroptosis-targeted therapy for diabetic kidney disease utilizing natural products. This approach holds significant promise for laying the experimental groundwork necessary for formulating new therapeutic strategies for chronic diseases associated with ferroptosis in the foreseeable future.
